# Characteristics of peptic ulcer bleeding in cirrhotic patients with esophageal and gastric varices

**DOI:** 10.1038/s41598-020-76530-3

**Published:** 2020-11-18

**Authors:** Zheng Lu, Xiaotian Sun, Jingjing Han, Bo Jin, Wenhui Zhang, Jun Han, Xuemei Ma, Bo Liu, Xiaoli Yu, Qin Wu, Yanling Wang, Hanwei Li

**Affiliations:** 1 Liver Cirrhosis Diagnosis and Treatment Center, The Fifth Medical Center of Chinese PLA General Hospital, Beijing, 100039 China; 2 Departement of Internal Medicine, Beijing South Medical District of Chinese PLA General Hospital, No. 1 North Liuli Bridge, Beijing, 100161 China

**Keywords:** Hepatology, Oesophagogastroscopy

## Abstract

Upper gastrointestinal bleeding (UGIB) is common in liver cirrhosis. Although esophageal and gastric varices (EGV) is the main bleeding source, there were still a proportion of patients with peptic ulcer bleeding. Thus, this study aimed to analyze the characteristic of variceal bleeding and peptic ulcer bleeding in liver cirrhosis. Cirrhotic patients with confirmed UGIB by urgent endoscopy from July 2012 to June 2018 were enrolled, and classified into peptic ulcer bleeding group (n = 248) and variceal bleeding group (n = 402). Clinical and endoscopic characteristics, therapeutic efficacy and prognosis were evaluated, and independent risk factors for 42-day morality were determined. The mean age and gender ratio of peptic ulcer bleeding group were higher than those in variceal bleeding group (55.58 ± 11.37 vs. 52.87 ± 11.57, *P* < 0.01; 4.51:1 vs. 2.87:1, *P* = 0.023). Variceal bleeding group most commonly presented as red blood emesis and coffee grounds (67.16%), while peptic ulcer group primarily manifested as melena (62.10%). Hepatocellular carcinoma was more prevalent in peptic ulcer group (141 vs. 119, *P* < 0.01). Albumin level in variceal bleeding group was lower higher (*P* < 0.01), but serum bilirubin, creatinine and prothrombin time were significantly higher (all *P* < 0.01). Success rate of endoscopic hemostasis for variceal bleeding and peptic ulcer bleeding was 89.05% and 94.35% (*P* = 0.021). Univariate and multivariate analysis identified prothrombin time (*P* = 0.041, OR [95% CI] 0.884 [0.786–0.995]), MELD score (*P* = 0.000, OR [95% CI] 1.153 [1.073–1.240]), emergency intervention (*P* = 0.002, OR [95% CI] 8.656 [2.219–33.764]), hepatic encephalopathy before bleeding (*P* = 0.003, OR [95% CI] 8.119 [2.084–31.637]) and hepatic renal syndrome before bleeding (*P* = 0.029, OR [95% CI] 3.877 [1.152–13.045]) as the independent predictors for 42-day mortality. Peptic ulcer bleeding should be distinguished from variceal bleeding by clinical and endoscopic characteristics.

## Introduction

Upper gastrointestinal bleeding (UGIB) is an important public health issue with a prevalence of 150 per 100 thousand each year^1,2^, and the inpatient mortality can be up to 10%^3^. In clinical practice, acute UGIB is a critical condition, and liver cirrhosis with UGIB is one of the deadliest complications in such patients. The main reasons included portal hypertension associated diseases like esophageal and gastric varices (EGV), portal hypertensive gastropathy (PHG) and other diseases observed in general population like peptic ulcer, acute gastric mucosal lesions (AGML), Mallory–Weiss syndrome and tumor. Among them, EGV is the most common cause for liver cirrhosis with UGIB. EGV accounts for 60–65% of cirrhotic patients with bleeding, a 6-week mortality can reach 20%^4^. The prognosis is closely correlated with the severity of liver diseases, and the effective and rapid treatment of acute UGIB can obviously reduce the mortality.

Although UGIB in cirrhotic patients with EGV is mostly caused by EGV, UGIB from peptic ulcer can also be detected in a relatively small proportion. Bleeding is one of the common and severe complications of peptic ulcer, and liver dysfunction and coagulation disorders will further increase the risk for UGIB in peptic ulcer patients. Previous studies have examined the prognostic effect of chronic liver diseases on the mortality of peptic ulcer with UGIB^5^, which reported that the 90-day mortality risk after admission in patients with chronic liver diseases, especially liver cirrhosis, was greatly higher than that in those without chronic liver diseases. The incidence and prevalence of peptic ulcer in patients with liver cirrhosis was increased^6,7^, but the possible pathogenesis has not been fully understood^8,9^. Siringoet al.^6^ prospectively evaluated the epidemiological and clinical features of 324 cirrhotic patients with peptic ulcer, and found that the prevalence and annual incidence of peptic ulcer was 11.7% and 4.3%, and over 70% of the patients were asymptomatic (no complaint of epigastric pain continuously over 7 days at diagnosis). Asymptomatic patients were even more in severe decompensated liver cirrhosis (*P* = 0.04). The clinical characteristics, endoscopic findings, treatment and prognosis of UGIB with peptic ulcer in cirrhotic patients are totally different from UGIB with EGV. However, UGIB with peptic ulcer in cirrhotic patients with EGV has been rarely studied. Thus, in this study we analyzed the features of UGIB with peptic ulcer in liver cirrhosis, and compared with UGIB with EGV. These results may help the clinicians to differentiate cirrhosis patients with UGIB and optimize the current therapeutic strategy.

## Methods

### Study design

Our hospital is a specialized hospital on liver disease and on-call urgent endoscopy (UE) has been performed since January 2010. Until December 31, 2018, a total of 12,491 cirrhotic patients with UGIB underwent UE. Among them, 8796 patients (70.42%) were diagnosed as UGIB, including 5338 EGV patients (42.73%). In this study, we retrospectively analyzed the data of 248 cirrhotic EGV patients with suspected UGIB who accepted UE within 2 h and confirmed as acute UGIB from peptic ulcer from July 2012 to June 2018 in our hospital (Peptic ulcer bleeding group). There were 402 patients diagnosed as active UGIB from EGV who were treated under endoscopy and had a second gastroscopy within 24–48 h (Variceal bleeding group). This study was approved by the Ethic Committee of the Fifth Medical Center of Chinese PLA General Hospital.

### Patients

From July 2012 to June 2018, all the cirrhotic patients have undergone UE within 2 h once UGIB was suspected. The inclusion criteria were (1) 18–80 years old; (2) clinical evidence of bleeding with 24 h including hematemesis and/or melena; (3) histologically diagnosed or clinical and radiographic data (ultrasound, CT, MRI) supporting liver cirrhosis; (4) drug administration at least 0.5 before UE; (5) stable vital signs like blood oxygen saturation > 90%, systolic blood pressure (SBP) ≥ 90 mmHg, diastolic blood pressure (DBP) ≥ 60 mmHg, controlled hepatic encephalopathy; (6) peptic ulcer as the only reason for bleeding in UE for peptic ulcer bleeding group and active UGIB from EGV and no other potential bleeding sources in primary UE for variceal bleeding group. Variceal bleeding group underwent endoscopic treatments in primary UE and had another endoscopic examination within 48 h, and further treatments would be administrated if necessary. Patients who were transferred from other hospitals were also included if they were eligible. The exclusions were (1) unsuitable for resuscitation; (2) UE performed in ICU; (3) history of bleeding wtthin two weeks or of endoscopic or interventional treatments or transjugular intrahepatic portosystem shunt (TIPS) within 1 week; (4) history of severe cardiovascular diseases, known allergy to terlipressin or sclerosing agent, pregnant; (5) bleeding from artificial ulcer created by endoscopic treatment; (6) bleeding from malignant ulcer, upper gastrointestinal tract tumors, Dieulafoy 's disease, PHG, duodenal venous varices, and other rare variceal bleeding.

Before UE, history taking, physical, ultrasound and laboratory examination were conducted, and all the patients were closely monitored. Blood pressure, heart rate and central venous pressure (CVP) were recorded every 2–4 h. Blood routine test, liver function, renal function and coagulation function were monitored. For patients with hypovolemia, red cells and fresh frozen serum were transfused to maintain hemoglobin ≥ 60 g/L. Variables included demographical factors (age, gender); causes for liver cirrhosis; history of bleeding, H. pylori infection, peptic ulcer and comprehensive treatments; vital signs (heart pressure, SBP, DBP, temperature, respiration rate); mental state; laboratory findings; H. pylori infection; and radiographic examinations. Baseline characteristics also included Child–Pugh score, MELD score, AIMS65 score, Rockall score, Glasgow-Blatch-ford score, UE findings, and the whole process of hemostasis.

### Urgent endoscopy

Hemostasis under UE was immediately performed after the patients gave their informed consent. All the procedures were completed by endoscopists with an experience of over 200 hemostatic interventions under UE. GIF-260 or GIF-290 endoscopy (Olympus, Tokyo, Japan) was used. Endoscopic findings of peptic ulcer bleeding were described by Forrest classification^10^. Common endoscopic treatments for active peptic ulcer bleeding included drug local injection (1:10,000 adrenalin sodium chloride solution) and clip (ANRY, China) closure^11^, and endoscopic treatments for variceal bleeding included ligation by 6-band ligator (COOK, the USA), sclerosing agent (10 ml lauryl gel or 2 ml sodium aluminate, China) injection and tissue gel injection. Tissue gel (Braun, Germany) was applied to endoscopically treat acute UGIB from isolated gastric varices (IGV) and gastroesophageal varices type 2 (GOV2)^12^. Successful endoscopic hemostasis was defined as no detection of active bleeding during withdrawal. Unsatisfactory endoscopic hemostasis was defined as unclear detection of active bleeding at the end of examinations. Failed endoscopic hemostasis was defined as ineffective treatment and the presence of active bleeding.

### Outcome definitions

All the patients were carefully monitored during admission and followed up at Day 42. The evidence of active bleeding was recorded. Hemoglobin and hematocrit were measured before and after the endoscopic procedures at least once at an interval of 2–5 days. Parameters evaluated were mode of treatment, volume of transfused blood, liver cirrhosis associated complications like spontaneous bacterial peritonitis (SBP), hepatic encephalopathy (HE), hepatic renal syndrome (HRS). Primary endpoints were 7-day and 42-day rebleeding rate, 5-day and 42-day mortality and the main death cause. Secondary endpoints were length of stay (LOS) and medical costs. The follow-ups were conducted by telephone or outpatient visit within 1 year. The repeated endoscopic findings, 1-year rebleeding rate, rebleeding diagnosis and liver cirrhosis related complications were all recorded, and the independent risk factors for 42-day mortality in cirrhotic EGV patients with acute UGIB from peptic ulcer were determined.

### Statistical analysis

All the statistical analysis was performed using SPSS software (version 23, IBM, Armonk, the USA). Contiguous data were presented as mean ± standard deviation (SD), and compared by independent t-test. Levene’s test was used for examining homogeneity of variance. Categorical data were presented as percentage (%), and compared by Pearson’s chi-square test or Fisher 's exact probability if applicable. Multivariate logistic regression model was applied to determine independent predictors for 42-day morality. Odd ratio (OR) and 95% confidence interval (CI) were calculated. *P* < 0.05 was considered to be statistically significant.

## Results

### Demographical and clinical characteristics

The mean age and gender ratio of peptic ulcer bleeding group were higher than those in variceal bleeding group (55.58 ± 11.37 vs. 52.87 ± 11.57, *P* < 0.01; 4.51:1 vs. 2.87:1, *P* = 0.023), but the proportion of patients admitted from emergency were lower (0.42:1 vs. 1.13:1, *P* < 0.01) (Table 1). Variceal bleeding group most commonly presented as red blood emesis and coffee grounds (67.16%), while peptic ulcer group primarily manifested as melena (62.10%). More patients in variceal bleeding group had a previous history of UGIB (212 vs. 80, *P* < 0.01), and were commonly treated by endoscopic therapy. Additionally, hepatocellular carcinoma was more prevalent in peptic ulcer group (141 vs. 119, *P* < 0.01).

**Table 1:** Demographical, clinical characteristics and laboratory findings.

Variables, n (%)	Variceal bleeding group (n = 402)	Peptic ulcer bleeding group (n = 248)	*P* value
**Age (years), mean ± SD**	52.87 ± 11.57	55.58 ± 11.37	< 0.01
**Sex, M/F**	298/104 (2.87:1)	203/45 (4.51:1)	0.023
**Admission status**			
Emergency Department/General Ward	213/189 (1.13:1)	73/175 (0.42:1)	< 0.01
**Liver cirrhosis etiology**			0.156
**Presentation**			< 0.01
Red blood emesis and Coffee grouds	270 (67.16%)	66 (26.61%)	< 0.01
Melena	106 (26.37%)	154 (62.10%)	< 0.01
Bloody stool	24 (5.97%)	25 (10.08%)	0.054
Other	2 (0.50%)	3 (1.21%)	0.313
**History of UGIB**	212 (52.74%)	80 (32.26%)	< 0.01
**History of bleeding treatment**			< 0.01
Pharmacologic therapy	57 (26.89%)	45 (56.25%)	< 0.01
Endoscopic therapy	94 (44.34%)	20 (25.00%)	< 0.01
TIPPS	8 (3.77%)	6 (7.50%)	0.714
Splenectomy or (+ endoscopic therapy)	50 (23.58%)	8 (10.00%)	< 0.01
Gastric coronary or splenic embolization	3 (1.42%)	0	0.173
Gastrectomy	0	1 (1.25%)	0.203
**Liver transplantation**	3 (0.75%)	1 (0.40%)	0.587
**Hepatocellular carcinoma**	141 (35.07%)	119 (47.98%)	< 0.01
**Vital signs, n (%)**			
SBP < 90 mmHg	40 (9.95%)	27 (10.89%)	< 0.01
SBP 90–100 mmHg	298 (74.13%)	36 (14.52%)	
SBP > 100 mmHg	64 (15.92%)	185 (74.60%)	
Heart rate > 100/min	233 (57.96%)	108 (43.55%)	< 0.01
**Lab test results, mean ± SD**			
Hemoglobin (131–172 g/dl)	82.38 ± 22.86	81.21 ± 24.25	0.533
Platelets (100–300 × 10^9^/l)	97.73 ± 66.09	93.43 ± 65.52	0.447
Albumin (35–55 g/l)	29.33 ± 5.51	26.64 ± 5.42	< 0.01
Bilirubin (3.4–20.5 umol/l)	48.71 ± 81.15	91.31 ± 114.10	< 0.01
Creatinine (62–115 umol/l)	88.63 ± 65.37	107.95 ± 83.90	< 0.01
Prothrombin time (11–14.3 s)	15.40 ± 3.96	16.57 ± 4.77	< 0.01
INR (0.8–1.2%)	1.38 ± 0.68	1.46 ± 0.72	0.119
Ascites, n (%) Medium-Large amount	97 (24.13%)	122 (49.19%)	< 0.01
Portal vein thrombosis, n (%)	130 (32.34%)	89 (35.89%)	0.352
**Collateral circulation, n (%)**			
None	243 (60.45%)	134 (54.03%)	< 0.01
Gastric-renal or spleen-renal shunt	42 (10.45%)	54 (21.77%)	
Para umbilical vein	117 (29.10%)	60 (24.19%)	
Child–Pugh score	7.84 ± 1.77	9.50 ± 2.08	< 0.01
Child–Pugh grade (A/B/C), n	106/233/63	19/114/115	< 0.01
MELD score	9.69 ± 7.10	13.49 ± 8.67	< 0.01
≥ 15, n (%)	71 (17.66%)	94 (37.90%)	< 0.01
AIMS65 score	1.14 ± 1.01	1.48 ± 1.01	< 0.01
≥ 2, n (%)	126 (31.34%)	120 (48.39%)	< 0.01
Rockall score	4.74 ± 1.56	4.41 ± 2.20	0.037
≥ 5, n (%)	172 (42.79%)	113 (45.56%)	0.488
Glasgow-Blatch-ford	13.49 ± 2.51	12.38 ± 3.57	< 0.01

We further analyzed the data of peptic ulcer group (Table 2). 119 patients (47.98%) underwent anti-tumor treatments including surgery, intervention and the like. 15.73% patients previously had peptic ulcer, 39.11% had a history of specific medications within 1 month, and 19.76% had surgical stress or trauma within 3 months. The ulcer was most commonly categorized as a < 1 cm (n = 136, 54.84%) Forrest classification III (n = 89, 35.89%) single lesion (n = 214, 86.29%) located in duodenal bulb and/or descending segment (n = 162, 65.32%). PPI was used in all the patients, and esophageogastroduodenoscopy within 3 months and within 3–6 months was repeated in 26 patients and 21 patients, respectively.

**Table 2:** Characteristics of peptic ulcer bleeding group.

Variables, n (%)	Peptic ulcer bleeding group (n = 248)
**History of HCC treatment**	119 (47.98%)
Surgery	3
Intervention	21
Radiotherapy	3
Radiofrequency	6
Argon-helium knife	3
Drug	50
Comprehensive treatment	33
**History of Peptic Ulcer**	39 (15.73%)
**History of HP (no test/negative/positive)**	169/60/19
**History of specific medications within 1 month**	97 (39.11%)
**History of surgical stress or trauma within 3 months**	49 (19.76%)
**Forrest classification**	
Ia	12 (4.84%)
Ib	32 (12.90%)
IIa	4 (1.61%)
IIb	44 (17.74%)
IIc	67 (27.02%)
III	89 (35.89%)
**Ulcer number**	
Single	214 (86.29%)
Multiple	34 (13.71%)
**Ulcer size**	
< 1 cm	136 (54.84%)
1–2 cm	61 (24.60%)
2––3 cm	18 (7.26%)
≥ 3 cm	24 (9.68%)
Unclear	10 (4.03%)
**Location**	
Fundus and/or body	6 (2.42%)
Augularis	16 (6.45%)
Antrum	44 (17.74%)
Multiple	2 (0.81%)
Pylorus	7 (2.82%)
Duodenal bulb and/or descending segment	162 (65.32%)
Stomach and duodenum	10 (4.03%)
Saddle after subtotal gastrectomy	1 (0.40%)
**Drug administration**	
Antibiotics	190 (76.61%)
Agent reducing portal pressure	193 (77.82%)
PPI	248 (100.00%)
***Helicobacter pylori***	
Positive/negative/no test	31/103/114
Esophagogastroduodenoscopy recheck within 3 months	26 (10.48%)
Esophagogastroduodenoscopy recheck within 3–6 months	21 (8.47%)

Stable vital signs were more common in peptic ulcer bleeding group than in variceal bleeding group. Albumin level in variceal bleeding group was higher (*P* < 0.01), but serum bilirubin, creatinine and prothrombin time were significantly lower (all *P* < 0.01). Ascites in medium-large amount (24.13% vs. 49.19%, *P* < 0.01) and gastric-renal or spleen-renal shunt (10.45% vs. 21.77%, *P* < 0.01) occurred more often in peptic ulcer group. Peptic ulcer bleeding group had higher Child–Pugh score (9.50 ± 2.08 vs. 7.84 ± 1.77), MELD score (13.49 ± 8.67 vs. 9.69 ± 7.10) and AIM65 score (1.48 ± 1.01 vs. 1.14 ± 1.01), but lower Rockall score (4.41 ± 2.20 vs. 4.74 ± 1.56) and Glasgow-Blatch-ford score (12.38 ± 3.57 vs. 13.56 ± 2.98) (all *P* < 0.01).

### UE findings

Under UE, severe esophageal varices (EV) (70.15% vs. 24.19%) and EGV (80.60% vs. 52.82%) were more common in variceal bleeding group (both *P* < 0.01) (Table 3). The primary source of bleeding was EV (n = 204, 50.75%), which was duodenal ulcer (DU, n = 162, 65.32%). The success rate of endoscopic hemostasis was 89.05% in variceal bleeding group and 94.35% in peptic ulcer bleeding, respectively (*P* = 0.021). For variceal bleeding, sclerosis and ligation was mostly used, while for peptic ulcer bleeding hemocoagulase spray was mostly used. The rate of unsatisfactory endoscopic hemostasis in variceal bleeding group was higher (6.72% vs. 2.42%, *P* = 0.015).

**Table 3:** Endoscopic findings.

Variables, n (%)	Variceal bleeding group (n = 402)		Peptic ulcer bleeding group (n = 248)		*P* value
**Esophageal varices**					
None	3 (0.75%)		8 (3.23%)		< 0.01
Mild	21 (5.22%)		94 (37.90%)		
Moderate	96 (23.88%)		86 (34.68%)		
Severe	282 (70.15%)		60 (24.19%)		
EBV	324 (80.60%)		131 (52.82%)		< 0.01
IGV	3 (0.75%)		8 (3.23%)		0.017
Source of bleeding	Esophageal varices	204 (50.75%)	Gastric ulcer	68 (27.42%)	–
	Cardiac part	133 (33.08%)	Duodenal ulcer	162 (65.32%)	
	Gastric varices	45 (11.19%)	Complex ulcer	10 (4.03%)	
	IGV	3 (7.46%)	Pylorus	7 (2.82%)	
	EV and GV	14 (3.48%)	Postoperative saddle	1 (0.40%)	
Successful endoscopic hemostasis	358/402 (89.05%)		234/248 (94.35%)		0.021
	Sclerosis/ligation	288 (71.64%)	Titanium clip	34 (13.71%)	–
	Tissue gel	56 (13.93%)	Drug injection	3 (1.21%)	
	Sclerosis + gel	14 (3.48%)	Hemocoagulase spray	197 (79.44%)	
Unsatisfactory endoscopic hemostasis	27 (6.72%)		6 (2.42%)		0.015
Failed endoscopic hemostasis	17 (4.23%)		8 (3.23%)		0.518
Sengstaken tube	22 (5.47%)		–		–
Emergency TIPS	2 (0.50%)		–		
Emergency intervention	–		17 (6.85%)		

### Outcomes

Obviously more patients in variceal bleeding group had blood transfusion (75.87% vs. 52.42%, *P* < 0.01), and more units of red blood cells were transfused (7.08 ± 7.00 vs. 7.32 ± 5.69, *P* < 0.01) (Table 4). The incidence of SBP and HRS immediately after bleeding in variceal bleeding group was lower than those in peptic ulcer group (1.74% vs. 6.05%, *P* < 0.01; 1.49% vs. 9.27%, *P* < 0.01). There were no statistically significant differences on rebleeding rate, morality, LOS and medical costs between two groups (all *P* > 0.05).

**Table 4:** Outcomes.

Variables, n (%)	Variceal bleeding group (n = 402)	Peptic ulcer bleeding group (n = 248)	*P* value
**Blood transfused**			
Red blood cells, n (%)	305 (75.87%)	130 (52.42%)	< 0.01
Units, median ± SD	7.32 ± 5.69	7.08 ± 7.00	< 0.01
Plasma, n (%)	148 (36.82%)	81 (32.66%)	0.281
Units, median ± SD	11.75 ± 15.78	19.38 ± 34.29	0.176
Platelet, n (%)	36 (8.96%)	14 (5.65%)	0.124
Units, median ± SD	2.39 ± 2.30	5.56 ± 9.21	0.469
**Complications of cirrhosis, n (%)**			
SBP before bleeding	38 (9.45%)	37 (14.92%)	0.034
SBP immediately after bleeding	7 (1.74%)	15 (6.05%)	< 0.01
SBP after bleeding in 42d	20 (4.98%)	14 (5.65%)	0.709
HE before bleeding	21 (5.22%)	19 (7.66%)	0.209
HE immediately after bleeding	18 (4.48%)	13 (5.24%)	0.657
HE after bleeding in 42d	18 (4.48%)	8 (3.23%)	0.429
HRS before bleeding	24 (5.97%)	20 (8.06%)	0.302
HRS immediately after bleeding	6 (1.49%)	23 (9.27%)	< 0.01
HRS after bleeding in 42d	11 (2.74%)	9 (3.63%)	0.522
**Rebleeding, n (%)**			
Within 7 days	54 (13.43%)	31 (12.50%)	0.742
8–42 days	30 (7.46%)	22 (8.87%)	0.520
**Mortality, n (%)**			
Within 42 d	29 (7.21%)	24 (9.68%)	0.265
Within 5 d	11 (2.74%)	11 (4.44%)	0.245
Within 6–42 d	18 (4.48%)	13 (5.24%)	0.657
**LOS, day, median ± SD**	20.36 ± 14.77	20.40 ± 18.12	0.975
**Medical costs, Yuan, median ± SD**	80,363.56 ± 70,373.08	78,215.60 ± 100,566.86	0.749

### Predictors of 42-day mortality

In order to identify the possible independent predictors for the mortality, we first examined the features of dead cases (Table 5). 29 patients in variceal bleeding group and 24 patients in peptic ulcer group died at 42-day follow-up. Hemoglobin level in variceal bleeding group was higher (71.88 ± 24.16 vs. 79.28 ± 22.25, *P* < 0.01), but Child–Pugh score (11.13 ± 2.17 vs. 8.93 ± 2.03, *P* < 0.01) and AIMS65 score (2.25 ± 0.94 vs. 1.69 ± 1.07, *P* < 0.05) were lower. There were more patients with confirmed hemostasis after UE in peptic ulcer bleeding (62.07% vs. 87.50%, *P* = 0.011). The most common death cause in variceal bleeding group was hemorrhagic shock (n = 20, 69.97%), but most of the patients in peptic ulcer group died of multiple systemic organ failure (MSOF, n = 12, 50.00%).

**Table 5:** Subgroup analysis of dead cases.

Variables, n (%)	Variceal bleeding group (n = 29)	Peptic ulcer bleeding group (n = 24)	*P* value
Age, years, mean ± SD	58.24 ± 14.59	55.67 ± 9.92	0.466
Sex, M/F	24/5 (4.80:1)	21/3 (7.00:1)	0.631
Hepatocellular carcinoma, n (%)	18 (62.07%)	15 (62.50%)	0.974
Hemoglobin (131–172 g/dl), mean ± SD	79.28 ± 22.25	71.88 ± 24.16	< 0.01
Platelets (100–300 × 10^9^/l), mean ± SD	102.38 ± 79.75	80.58 ± 53.02	0.055
Albumin (35–55 g/l), mean ± SD	27.48 ± 5.34	24.83 ± 5.08	0.051
Bilirubin (3.4–20.5 umol/l), mean ± SD	117.77 ± 156.77	183.97 ± 176.49	0.225
Creatinine (62–115 umol/l), mean ± SD	114.62 ± 74.27	168.21 ± 125.36	0.140
Prothrombin time (11–14.3 s), mean ± SD	18.32 ± 8.47	19.38 ± 5.83	0.066
INR (0.8–1.2%), mean ± SD	1.60 ± 0.74	1.73 ± 0.57	0.479
Child–Pugh score, mean ± SD	8.93 ± 2.03	11.13 ± 2.17	< 0.01
MELD score, mean ± SD	16.35 ± 9.55	21.88 ± 11.07	0.055
AIMS65 score, mean ± SD	1.69 ± 1.07	2.25 ± 0.94	0.049
Rockall score, mean ± SD	5.41 ± 1.82	6.08 ± 2.15	0.225
Glasgow-Blatch-ford, mean ± SD	14.31 ± 2.52	15.50 ± 3.26	0.140
Confirmed hemostasis after UE, n (%)	16 (62.07%)	21 (87.50%)	0.011
Other emergent hemostatic treatments, n (%)	7 (24.14%)*	5 (20.83%)^#^	0.775
Rebleeding within 42d, n (%)	4 (13.79%)	7 (29.17%)	0.170
Transfused red blood, n (%)	25 (86.21%)	19 (79.17%)	0.497
Units of red blood, mean ± SD	9.55 ± 7.94	6.42 ± 6.85	0.135
Transfused plasma, n (%)	19 (65.52%)	15 (62.50%)	0.820
Units of Plasma, mean ± SD	12.59 ± 20.71	10.00 ± 17.24	0.627
LOS, days, mean ± SD	14.31 ± 11.03	18.54 ± 16.61	0.273
Costs, yuan, mean ± SD	102,662.00 ± 89,616.75	101,979.46 ± 98,881.80	0.979
Time from bleeding to death, days, mean ± SD	8.03 ± 9.92 (2–33)	8.96 ± 9.51 (1–39)	0.732
**Cause of death, n (%)**			
Hemorrhagic shock	20 (69.97%)	4 (16.67%)	< 0.01
Septic shock	3 (10.34%)	3 (12.50%)	0.805
MSOF	5 (17.24%)	12 (50.00%)	0.011
Bleeding from ruptured liver carcinoma and liver failure	0	4 (16.67%)	–
Brain hernia	1 (3.45%)	1 (4.17%)	1.000

*Sengstaken tube.

^#^Intervertion.

A total of 46 variables were included for univariate analysis in predicting the outcome of peptic ulcer bleeding in cirrhotic patients with EGV (Table 6). 20 factors were shown to be potential predictors of 42-day mortality. Multivariate logistic regression model further identified prothrombin time (*P* = 0.041, OR [95% CI] 0.884 [0.786–0.995]), MELD score (*P* = 0.000, OR [95% CI] 1.153 [1.073–1.240]), emergency intervention (*P* = 0.002, OR [95% CI] 8.656 [2.219–33.764]), hepatic encephalopathy before bleeding (*P* = 0.003, OR [95% CI] 8.119 [2.084–31.637]) and hepatic renal syndrome before bleeding (*P* = 0.029, OR [95% CI] 3.877 [1.152–13.045]) as the independent predictors.

**Table 6:** Univariable analysis and multivariable logistic regression to determine factors associated with 42-day mortality for peptic ulcer bleeding in cirrhotic patients with GOV.

Variables, n (%)	Alive (n = 224)	Dead (n = 24)	Univariable analysis	Multivariable analysis		
			*P* value	*P* value	OR	95% CI
Age, years, mean ± SD	55.57 ± 11.54	55.67 ± 9.92	0.967			
Sex, M/F	182/42 (4.33:1)	21/3 (7.00:1)	0.454			
Hepatocellular carcinoma, n (%)	104 (46.43%)	15 (62.50%)	0.520			
History of HCC treatment, n (%)	62 (27.68%)	7 (29.17%)	0.929			
History of UGIB, n (%)	75 (33.48%)	5 (20.83%)	0.214			
History of positive HP infection, n (%)	18 (8.04%)	1 (4.17%)	0.506			
History of Peptic Ulcer, n (%)	38 (16.96%)	1 (4.17%)	0.321			
History of special drug, n (%)	85 (37.95%)	12 (50.00%)	0.254			
History of stress trauma, n (%)	44 (19.64%)	5 (20.93%)	0.889			
Collateral circulation, n (%)	104 (46.43%)	10 (41.67%)	0.657			
Portal thrombosis, n (%)	79 (35.27%)	10 (41.67%)	0.535			
Ascites (medium to large), n (%)	106 (47.32%)	16 (66.67%)	0.077			
Hemoglobin, mean ± SD	82.21 ± 24.10	71.88 ± 24.16	0.049			
Platelets, mean ± SD	97.80 ± 67.00	80.58 ± 53.02	0.316			
Albumin, mean ± SD	26.84 ± 5.43	24.83 ± 5.79	0.087			
Bilirubin, mean ± SD	81.39 ± 100.92	183.97 + 176.49	0.000			
Creatinine, mean ± SD	101.50 ± 75.79	168.21 ± 125.36	0.005			
PT, mean ± SD	16.27 ± 4.55	19.38 ± 5.83	0.008	0.041	0.884	0.786–0.995
INR, mean ± SD	1.43 ± 0.73	1.73 ± 0.57	0.137			
Child–Pugh score, mean ± SD	9.33 ± 2.00	11.13 ± 2.17	0.000			
MELD score, mean ± SD	12.59 ± 7.89	21.88 ± 11.07	0.000	0.000	1.153	1.073–1.240
AIMS65 score, mean ± SD	1.40 ± 0.98	2.25 ± 0.94	0.000			
Rockall score, mean ± SD	4.23 ± 2.13	6.08 ± 2.15	0.000			
Glasgow-Blatch-ford score, mean ± SD	12.04 ± 3.45	15.50 ± 3.26	0.000			
Esophageal varices (media-severe), n (%)	133 (59.38%)	13 (54.17%)	0.871			
Esophageal and Gastric varices, n (%)	124 (55.36%)	7 (29.17%)	0.019			
Source of bleeding (GU/PU/others), n	61/147/16	7/15/2	0.949			
Forrest classification, n (%) (Ia/Ib/IIa)	41 (18.30%) (11/27/3)	7 (29.17%) (1/5/1)	0.228			
Forrest classification IIb, n (%)	36 (16.07%)	7 (29.17%)	0.114			
Forrest classification IIc/III, n (%)	146 (65.18%) (62/84)	10 (41.67%) (5/5)	0.025			
Successful endoscopic hemostasis, n (%)	33 (14.73%)	4 (16.67%)	0.706			
Unsatisfactory endoscopic hemostasis, n (%)	5 (2.23%)	1 (4.17%)	0.075			
Failed endoscopic hemostasis and other treatments suggested, n (%)	6 (2.68%)	2 (8.33%)	0.340			
Emergency intervention, n (%)	12 (5.36%)	5 (20.83%)	0.008	0.002	8.656	2.219–33.764
Rebleeding within 7 days, n (%)	28 (12.50%)	3 (12.50%)	0.949			
Rebleeding 8–42 days, n (%)	18 (8.04%)	4 (16.67%)	0.168			
Transfused red blood cells, n (%)	111 (49.55%)	19 (79.17%)	0.009			
Units of transfused red blood cells, mean ± SD	3.42 ± 5.93	6.42 ± 6.85	0.033			
Transfused plasma, n (%)	66 (29.46%)	15 (62.50%)	0.002			
Units of transfused plasm, mean ± SD	6.05 ± 21.94	10.00 ± 17.24	0.397			
SBP before bleeding, n (%)	29 (12.95%)	8 (33.33%)	0.011			
SBP immediately after bleeding, n (%)	13 (5.80%)	2 (8.33%)	0.623			
HE before bleeding, n (%)	12 (5.36%)	7 (29.17%)	0.000	0.003	8.119	2.084–31.637
HE immediately after bleeding, n (%)	10 (4.46%)	3 (12.50%)	0.016			
HRS before bleeding, n (%)	12 (5.36%)	8 (33.33%)	0.000	0.029	3.877	1.152–13.045
HRS immediately after bleeding, n (%)	18 (8.04%)	5 (20.83%)	0.049			

### Ethics statement

All methods followed were carried out in accordance with the ethical standards of the responsible committee on human experimentation (institutional and national) and with relevant guidelines and regulations and the Helsinki Declaration of 1975, as revised in 2008 (5). Informed consent was obtained from all patients for being included in the study.

## Discussion

At present, it is very difficult to differentiate the cause of UGIB in cirrhotic patients. For such patients, variceal bleeding is usually suspected and the possibility of peptic ulcer bleeding is easily neglected. However, it may be unsuitable to administrate specific treatments before determining the cause of UGIB. Thus, in this paper we analyzed and compared the clinical characteristics, endoscopic findings, treatments and prognosis of variceal bleeding and peptic ulcer bleeding in cirrhotic patients.

Peptic ulcer bleeding patients were prone to be old and male, and the age was older than that in variceal bleeding group and that in general population with peptic ulcer previously reported^13,14^. Variceal bleeding occurs more urgent than peptic ulcer bleeding, supported by the facts that more patients in variceal bleeding group had previous history of UGIB, endoscopic treatment and surgery and the chief symptom was hematemesis.

A retrospective study showed that the positivity of H. pylori infection in cirrhotic patients with peptic ulcer was greatly lower than 90% in duodenal ulcer patients and 70–80% in gastric ulcer patients^15^. The pathogenesis of peptic ulcer in liver cirrhosis remains unclear. Our findings indicated that H. pylori infection may be not the main reason for peptic ulcer, while drug, psychological factors and surgical trauma could contribute more. So, whether the patients had previous history of peptic ulcer or H. pylori infection could not provide useful information in judging the presence of peptic ulcer in cirrhotic patients. Nearly all the patients take at least three kinds of different drugs for a long time, and hepatocellular carcinoma patients receive surgery, intervention, radiotherapy and comprehensive treatment, which all participate in the development of peptic ulcer together with coagulation dysfunction. In China, Chinese herbs and their derivatives have been widely applied in the treatment of liver cirrhosis. Some herbs could affect the normal barrier function of gastric mucosa, and synergize with non-steroid anti-inflammation drugs in causing peptic ulcer^16^.

Peptic ulcer bleeding group had worse liver reserve function, and the higher MELD score, which predicted a worse outcome. The potential reasons for that may be that more patients in PUB group had progressive liver cancer and multiple organ injuries and their total bilirubin and prothrombin time was higher, while more patients in variceal bleeding group had ever received specific prevention or treatment due to bleeding. More collateral circulation was observed in PUB group, possibly indicating that the presence of collateral circulation could reduce the portal hypertension and the bleeding risk. Although international guidelines recommend that risk factors should be evaluated for acute UGIB patients^17,18^, no specialized scoring system for acute UGIB in liver cirrhosis patients is available right now. In our study, AIMS65, Rockall and Glasgow-Blatch-ford scores were not proved to be independent risk factors for 42-day morality. AIMS65 has 5 variables^19^, and it is mainly used to evaluate the mortality of acute UGIB. AIMS65 score and the percentage of patients with AIMS65 ≥ 2 in peptic ulcer bleeding group were obviously higher than those in variceal bleeding group, which may be explained by the even lower hemoglobin and worse coagulation function in peptic ulcer bleeding group. Rockall score combined clinical data and endoscopic parameters, which can categorize the patients into high risk (≥ 5), moderate risk and low risk according to their age, shock status, comorbidity, endoscopic diagnosis and active bleeding under endoscopy^20^. Statistical analysis showed that mean Rockall score in peptic ulcer bleeding group was lower than that in variceal bleeding group, and the possible reason may be the higher incidence of low blood pressure and tachycardia in variceal bleeding group. Glasgow-Blatch-ford score is mainly used in predicting whether acute UGIB patients need transfusion, endoscopic treatment and surgery or not, and its most distinguishing feature is to identify those with low risk who do not require admission^21^. Hemoglobulin and blood urea share a large proportion of Glasgow-Blatch-ford score, which was inappropriate for liver cirrhosis, because a large majority of decompensate cirrhotic patients have moderate anemia and renal dysfunction. Of 650 cirrhotic patients in this study, 646 patients (99.38%) had Glasgow-Blatch-ford score ≥ 6, which is set as high risk in previous literature and guidelines. This may be of no value in stratifying UGIB in liver cirrhosis. Furthermore, AIMS65, Rockall and Glasgow-Blatch-ford score predicted a worse prognosis in peptic ulcer bleeding group, but there were no significant differences on 42-day rebleeding rate and mortality. This could suggest that the value of current international evaluations on rebleeding and mortality for acute UGIB is quite limited in liver cirrhosis with EGV. Our future research will aim to establish a tool to stratify the bleeding risk in such patients and predict the prognosis, which can be used to guide the treatment and reduce the mortality.

There were 17.74% patients with Forrest classification Ia and Ib and 1.6% with Forrest IIa, which was different from 12 and 8% in previous report^22^; but the total percentage of Forrest Ia, Ib and IIa was comparable (19.35% vs. 20%). The worse coagulation function and lower platelet count in cirrhotic patients with peptic ulcer bleeding may partly explain it. Thus, it is common that active bleeding from naked vessels is often observed in such patients and less Forrest IIa patiens were detected. All the patients with high risk in received endoscopic interventions and the success rate of endoscopic hemostasis was up to 75.00%. The overall success rate of endoscopic hemostasis for 92 patients with Forrest Ia, Ib, IIa and IIb was 84.78%. The main reason for early rebleeding in liver cirrhosis with peptic ulcer was the rebleeding of ulcer, while about a half of the late rebleeding was due to portal hypertension related bleeding. Forrest IIb peptic ulcer in liver cirrhosis was potentially with high risk, and its rebleeding rate can be as high as high risk (Forrest Ia, Ib and IIa). It is highly recommended that complete washing and endoscopic intervention as soon as possible should reduce the rebleeding risk. Although peptic ulcer bleeding was not manifested as severe and urgent as variceal bleeding, there was no statistical difference on success rate of endoscopic hemostasis, LOS, medical costs and 42-day mortality between them.

Proper attention has not been paid to the prevention and treatment of cirrhotic patients with peptic ulcer in current practice. One reason was that the application of proton pump inhibitors (PPI) in liver cirrhosis was still on doubt^23–26^, and the other reason was that the antibiotics for eradicating *H. pylori* may aggravate liver injury, especially for decompensated liver cirrhosis patients. Some studies proved that PPI did not increase the incidence of SBP^27^, while other studies verified that PPI can induce SBP^28^. However, most of the studies did not take the PPI dosage into consideration, and the pharmacological function of PPI is dosage dependent. Our study focused on the correlation of PPI with SBP and HE, especially the effects of high dosage PPI on the occurrence of SBP and HE in treating peptic ulcer bleeding in patients with decompensated liver cirrhosis. The findings indicated that the short-term usage of PPI did not increase the risk for liver cirrhosis related complications, and it still needs to be validated in prospective trials. There was also limitation. Our retrospective study can only support that PPI was safe and effective as an adjuvant treatment for liver cirrhosis, but it could not be able to investigate the effects of PPI on the development of SBP, HE and HRS, which should be further clarified in future designed researches as well as the potential dosage and time dependent risk.

Seen by this, we highly suggest that the prevention of peptic ulcer should be an important part of comprehensive treatment for liver cirrhosis with hepatocellular carcinoma. Peptic ulcer bleeding is suspected, if UGIB is observed in cirrhotic patients who recently have stress, trauma, surgery or special drug treatment.
